# Smartphone apps to support laypersons in bystander CPR are of ambivalent benefit: a controlled trial using medical simulation

**DOI:** 10.1186/s13049-021-00893-3

**Published:** 2021-06-03

**Authors:** Camilla Metelmann, Bibiana Metelmann, Louisa Schuffert, Klaus Hahnenkamp, Marcus Vollmer, Peter Brinkrolf

**Affiliations:** 1 Department of Anaesthesiology, University Medicine Greifswald, Greifswald, Germany; 2 Institute of Bioinformatics, University Medicine Greifswald, Greifswald, Germany

**Keywords:** resuscitation, cardiac arrest, teaching, health informatics, mHealth, smartphone

## Abstract

**Background:**

Bystander-initiated resuscitation is essential for surviving out-of-hospital cardiac arrest. Smartphone apps can provide real-time guidance for medical laypersons in these situations. Are these apps a beneficial addition to traditional resuscitation training?

**Methods:**

In this controlled trial, we assessed the impact of app use on the quality of resuscitation (hands-off time, assessment of the patient’s condition, quality of chest compression, body and arm positioning). Pupils who have previously undergone a standardised resuscitation training, encountered a simulated cardiac arrest either (i) without an app (control group); (ii) with facultative app usage; or (iii) with mandatory app usage. Measurements were compared using generalised linear regression.

**Results:**

200 pupils attended this study with 74 pupils in control group, 65 in facultative group and 61 in mandatory group. Participants who had to use the app significantly delayed the check for breathing, call for help, and first compression, leading to longer total hands-off time. Hands-off time during chest compression did not differ significantly. The percentage of correct compression rate and correct compression depth was significantly higher when app use was mandatory. Assessment of the patient’s condition, and body and arm positioning did not differ.

**Conclusions:**

Smartphone apps offering real-time guidance in resuscitation can improve the quality of chest compression but may also delay the start of resuscitation. Provided that the app gives easy-to-implement, guideline-compliant instructions and that the user is familiar with its operation, we recommend smartphone-guidance as an additional tool to hands-on CPR-training to increase the prevalence and quality of bystander-initiated CPR.

**Supplementary Information:**

The online version contains supplementary material available at 10.1186/s13049-021-00893-3.

## Introduction

Treatment of out-of-hospital cardiac arrest has to start as early as possible [1]: A bystander-initiated cardiopulmonary resuscitation (CPR) can substantially increase the likelihood of survival [2–4] - even when performed by medical laypersons [5, 6]. Both the European Resuscitation Council and the American Heart Association promote bystander CPR [7–9]. However, although most out-of-hospital cardiac arrests are witnessed, rates of bystander CPR remain low, with high variance between European countries [10–13]. One of the impediments why medical laypersons do not start CPR is limited knowledge [14–16] – a factor particularly affecting population groups without access to in-person first aid courses [17]. To bridge this gap, a multitude of different teaching concepts has been implemented resulting in higher rates of bystander CPR [18–21]. Smartphone applications (apps) can help impart sufficient knowledge about resuscitation [22–24], and provide step-by-step guidance to laypersons encountering out-of-hospital cardiac arrest [25]. The vast majority of the population has a smartphone at hand at all times and is familiar with its operation. It seems reasonable to use this resource to enable medical laypersons to perform first aid procedures more confidently [26], and ultimately to increase the prevalence of bystander-initiated CPR. The European Resuscitation Council has been recommending the use of mobile devices in CPR since 2015 [27, 28] and the American Heart Association calls for further studies analysing mobile devices to facilitate CPR [29].

The benefits of using an app during this time-sensitive endeavour have to outweigh possible disadvantages. We aimed to analyse whether a high-quality smartphone-app offering real-time guidance (added to a previous CPR training) increases the quality of bystander CPR by medical laypersons.

## Methods

### Aim, design, setting of the study

In this controlled trial we used a standardised simulated cardiac arrest scenario to study the impact of app use on the quality of resuscitation, as defined by the following criteria: time until check for breathing; time until call for help; time until first chest compression; assessment of consciousness, airway obstruction and breathing; chest compression rate; depth of chest compressions; release of chest compression; hands-off time during chest compression; hand positioning; body positioning; arm positioning.

After (i) a systematic review of available apps guiding a medical layperson through a resuscitation situation, (ii) an adherence-testing to medical guidelines and (iii) a usability evaluation [30] the smartphone app “HELP Notfall” (Schweizerische Herzstiftung, version 1.0) was assessed to be “best suitable” for our study. The app provides step-by-step guidance with visual and acoustic instructions.

**Fig. 1:**
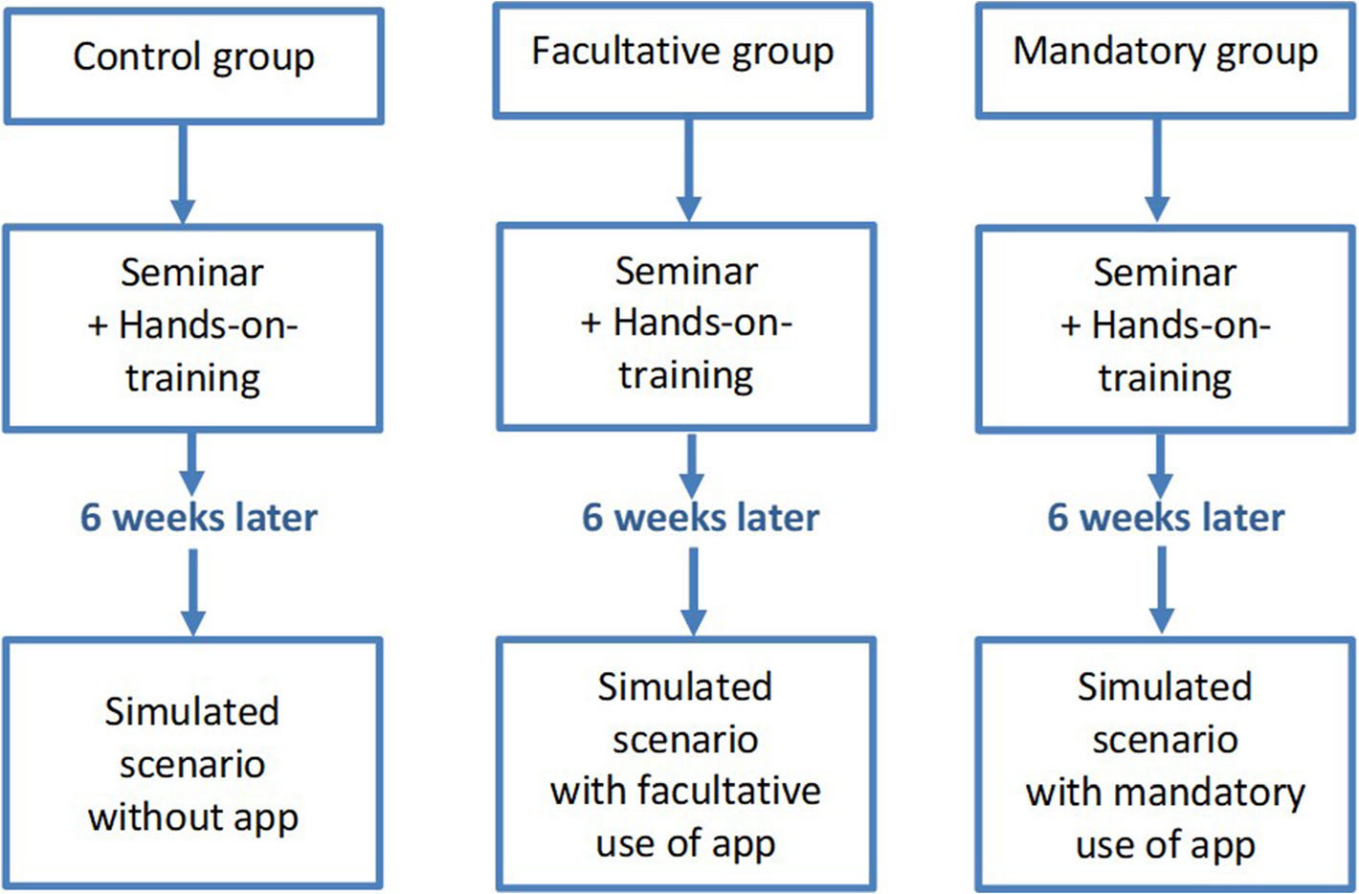
Study design

Participants were divided into three groups: in the “mandatory group” the app had to be used during the scenario; in the “facultative group” app use was at the discretion of the study participant; in the control group no smartphone use was allowed (Fig. 1). All participants received a standardised 30-minute seminar on one-person hands-only-CPR (in groups of up to 25 participants) based on the European Resuscitation Council guidelines 2015 (detecting cardiac arrest, calling for help, high quality chest compression) [31]. All seminars were held by the same lecturer (LS), a senior medical student and experienced paramedic. In all groups, seminars were followed by hands-on-training using manikins that provide real-time feedback. During this one-on-one training each participant also received individual feedback from the instructor. Participants in the mandatory group were introduced to the app “HELP Notfall” and operation was demonstrated and practised. Participants in the facultative and mandatory group were encouraged to download the app after the seminar to familiarize themselves with it. Participants in the control group were not informed about the app but provided with a fact sheet summarizing the seminar.

Six weeks after the seminar and hands-on-training, resuscitation skills were assessed in a standardised scenario (collapsed person in a park). All participants encountered, detected and treated a simulated cardiac arrest individually. Quality of resuscitation was assessed using the simulation manikin Brayden® (Innosonian®, South Korea). The manikin software records information on chest compression rate, depth, release, fraction and hand position. Quality of resuscitation was also evaluated based on time measurements, the detection of unconsciousness, the assessment of breathing and the body position during chest compression (a translated version of the score chart is provided as E-Supplement 1). Participants of the control group entered the scenario without a smartphone; participants of the facultative group were allowed to use the app “HELP Notfall” on a voluntary basis; participants of the mandatory group were instructed to use the app.

### Participants’ characteristics

To conduct this study, we obtained permission by the education authority (Ministry of Education, Science and Culture of Mecklenburg-Vorpommern, Germany) and contacted headteachers of all five secondary schools (German “Gymnasium”) in two cities; four of these agreed to participate. Gymnasium is the highest of the three main tracks of the secondary school system in Germany, the diploma “Abitur” after the 12th grade allows students access to university. All participants were pupils attending class 8 to 11. The seminar and the resuscitation assessment were conducted in the classrooms as part of a school lesson. Although seminar and hands-on-training were obligatory, study participation was voluntary. Written consent to participate was obtained from all participants and their parents or legal guardians prior to the study.

Allocation into the three groups was done per school to minimize contact between different study groups. The aim was to reduce the risk of pupils in control group downloading the app, because they were told about it in the schoolyard by pupils of the other two groups. Pupils in the mandatory group were from two different schools (Gymnasium 3 and 4), because the number of pupils in these schools were smaller compared to Gymnasium 1 and 2. The study was carried out between October 2017 and July 2018.

### Statistical Analysis

Primary outcome of our study was total hands-off time (time until first compression + time of all pauses during CPR). Power analysis was done using the software G*Power (Universität Düsseldorf, Mannheim, Kiel; Germany) and was based on a study assessing no-flow-time comparing layperson-CPR with and without telephone guidance [32]. An effect size of 0.83 was calculated. We decided on a power of 0.9 and a type I error probability of 0.05. To account for dropouts, more than one school class per group was recruited. Statistical processing of the data was carried out using IBM SPSS Statistics, version 25.0 (IBM Corporation, Armonk, New York, USA) and Microsoft Excel 2010 (Microsoft Corporation, Redmond, Washington, USA). Generalised linear regression on 1,000 bootstrap samples was performed for the dependent variables “times”, “percentage of correct compression rate”, “percentage of correct compression depth”, “percentage of correct release of compression”, and “percentage of correct hand position”. As independent variables “group”, “gender”, “age”, “pre-existing first aid knowledge”, and “previous encounter of emergency situation” were chosen to consider main influencing factors as well as to adjust for differences between the groups.

## Results

200 pupils attended this study with 74 participants (37 %) in control group, 65 participants (32,5 %) in facultative group and 61 participants (30,5 %) in mandatory group. Characteristics of study participants are depicted in Table 1. Mean age was lower in the facultative group (13.9 years), than in control group (14.6 years) and mandatory group (15.3 years). Most students had some knowledge about first aid procedures prior to attending the seminar but had not been in an emergency situation before.

**Table 1:** Characteristics of study participants

	Total (n,%)	Control group (n,%)	Facultative group (n,%)	Mandatory group (n,%)
**Number of participants**	200	74 (37.0)	65 (32.5)	61 (30.5)
**Gender**	197	71	65	61
Male	95 (48.2)	37 (52.1)	31 (47.7)	27 (44.3)
Female	102 (51.8)	34 (47.9)	34 (52.3)	34 (55.7)
**Age (years)**	196	71	64	61
13	37 (18.9)	14 (19.7)	23 (35.9)	0
14	53 (27.0)	21 (29.6)	24 (37.5)	8 (13.1)
15	57 (29.1)	16 (22.5)	10 (15.6)	31 (50.8)
16	32 (16.3)	10 (14.1)	7 (10.9)	15 (24.6)
17	17 (8.7)	10 (14.1)	0	7 (11.5)
**Pre-existing first aid knowledge**^a^	192	77	63	52
None	71 (37.0)	29 (37.7)	19 (30.1)	23 (44.3)
First Aid Course	105 (54.7)	37 (48.1)	42 (66.7)	26 (50.0)
Member of volunteer fire brigade	5 (2.6)	4 (5.2)	1 (1.6)	0
School paramedic	4 (2.1)	3 (3.8)	0	1 (1.9)
Other	7 (3.6)	4 (5.2)	1 (1.6)	2 (3.8)
**Have you been in an emergency situation before?**	196	74	61	61
Yes	12 (6.1)	3 (4.1)	5 (8.2)	4 (6.6)
No	184 (93.9)	71 (95.9)	56 (91.8)	57 (93.4)

^a^ Legend: multiple answers possible

Half of the participants in the facultative group decided to use the app.

**Fig. 2:**
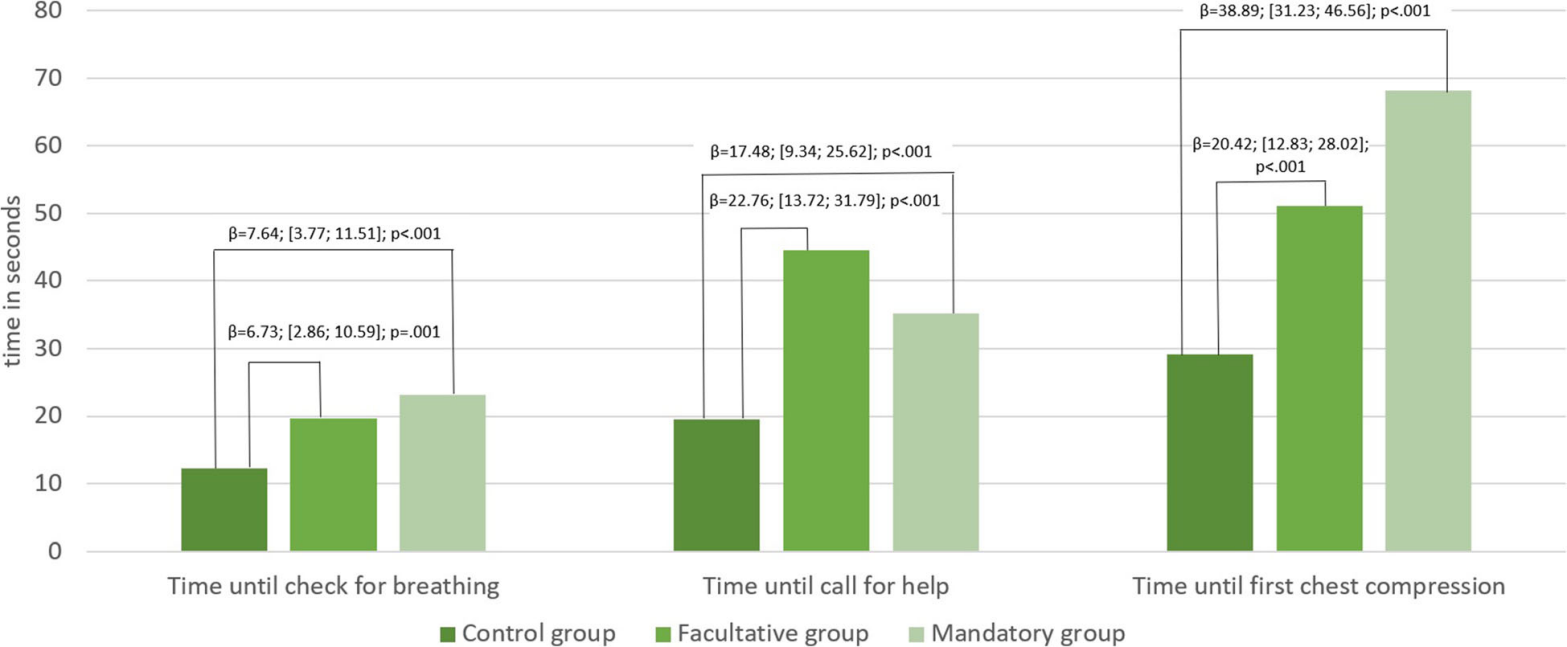
Average measured time intervals during CPR simulation with differences (beta with 95 % confidence interval) adjusted for gender, age, pre-existing first aid knowledge, and previous encounter of emergency situation

The average measured time intervals differed significantly between the groups (Fig. 2 and E-Table 1). In the groups that used the app (facultative group, mandatory group), the call for help and the first chest compression were significantly delayed compared with the control group (*p* < .001). When app use was mandatory, the participants also took significantly more time to check for breathing compared with the control group (*p* < .001). Assessment of breathing was done in similar time spans in all groups.

Hands-off time during compression was a total of 1.5 s when participants did not have the chance to use the app (control group), and 11.0 s when app use was facultative (multivariable adjusted effect: β = 9.73 s; 95 %-CI: [5.52; 13.93]; *p* < .001). Participants with mandatory use of app had a hands-off time of 0.5 s during compression, which was not significantly different to the control group (β=-1.06 s; 95 %-CI: [-5.29; 3.17]; *p* = .62). The total hands-off time (time until first compression + time of all pauses during compression) was significantly longer in the facultative group (β = 29.22 s; 95 %-CI: [20.79; 37.65]; *p* < .001) and in the mandatory group (β = 38.02 s; 95 %-CI: [29.55; 46.50]; *p* < .001) than in the control group.

Regardless of app use, the majority of participants in all study groups assessed consciousness, airway obstruction and breathing in the correct way (Table 2).

**Table 2:** Assessment of consciousness, airway obstruction and breathing

	Control group (n,%)	Facultative group (n,%)	Mandatory group (n,%)
**Assessment of consciousness**	*n* = 74	*n* = 64	*n* = 60
Not checked	5 (6.8 %)	2 (3.1 %)	3 (5.0 %)
Spoke to **or** touched person	2 (2.7 %)	5 (7.8 %)	4 (6.7 %)
Spoke to **and** touched person	67 (90.5 %)	57 (89.1 %)	53 (88.3 %)
**Assessment of airway obstruction**	*n* = 74	*n* = 63^a^	*n* = 60
Not checked	24 (32.4 %)	19 (30.2 %)	15 (25.0 %)
Checked, but not correctly	9 (12.2 %)	13 (20.6 %)	12 (20.0 %)
Correctly checked	41 (55.4 %)	31 (49.2 %)	33 (55.0 %)
**Assessment of breathing**	*n* = 74	*n* = 64	*n* = 60
Not checked	7 (9.5 %)	4 (6.2 %)	0
1 out of look, listen, feel	5 (6.8 %)	5 (7.8 %)	10 (16.7 %)
2 out of look, listen, feel	14 (18.8 %)	20 (31.3 %)	12 (20.0 %)
3 out of look, listen, feel	48 (64.9 %)	35 (54.7 %)	38 (63.3 %)
^a^ Legend: difference caused by loss of data			

All but 2 participants performed chest compressions. When app use was mandatory, the percentage of chest compressions with the correct compression rate and recommended depth was significantly higher than when no app was available to use (Table 3). There were no significant differences regarding release of chest compression and hand positioning between the mandatory group and the control group.

**Table 3:** Quality of chest compression with adjusted group effects

	Control group	Facultative group	Mandatory group	Control vs. facultative group β [95 %CI]; p	Control vs. mandatory group β [95 %CI]; p
**Correct compression rate [100–120/min**^**− 1**^**] (%)**				β=-4.59 [-16.91; 7.73]; *p* = .47	β = 18.61 [6.10; 31.11]; *p* = .004
mean (standard deviation)	43.8 (±36.8)	39.2 (±34.8)	65.4 (±32.7)		
median (lower; upper quartile)	42 (6; 81)	32 (6.3; 74.8)	81 (44.3; 89)		
**Correct compression depth [5-6 cm] (%)**				β = 11.20 [-0.57; 22.98]; *p* = .06	β = 22.91 [10.97; 34.86]; *p* < .001
mean (standard deviation)	24.4 (±30.4)	30.1 (±35.2)	47.6 (±37.3)		
median (lower; upper quartile)	10 (0.5; 36.5)	7.5 (0; 59.5)	51.5 (9.3; 82.5)		
**Correct release of compression (%)**				β=-10.46 [-19.72; -1.20] ; *p* = .027	β = 1.34 [-7.99; 10.66]; *p* = .78
mean (standard deviation)	89.7 (±22.6)	81.7 (±30.9)	87.8 (±23.8)		
median (lower; upper quartile)	100 (94.6; 100)	98 (77.5; 100)	100 (89.3; 100)		
**Correct hand position (%)**				β=-1.29 [-10.64; 8.06]; *p* = .79	β = 6.13 [-3.28; 15.54]; *p* = .20
mean (standard deviation)	76.8 (±28.3)	77.6 (±30.9)	86.4 (±19.0)		
median (lower; upper quartile)	90 (64.3; 99)	95 (59.3; 99)	97 (79.3; 100)		

The majority of participants showed a correct body position and arm posture during the chest compressions (Table 4). All participants in the mandatory group had a correct body position.

**Table 4:** Positioning of first aider during chest compressions

	Control group (n,%)	Facultative group (n,%)	Mandatory group (n,%)
Correct body position	63 (85.1)	61 (95.4)	60 (100)
Correct arm posture	72 (97.3)	60 (93.7)	57 (95.0)

## Discussion

We found that real-time guidance by a high-quality smartphone app helped medical laypersons to correctly perform some aspects of CPR; but it also led to a higher total hands-off time and a delayed start of chest compressions.

Our study shows the positive and negative effects of app use on resuscitation at a level of detail that goes beyond the extent of the studies published to date. Prior to the simulated cardiac arrest scenario, all participants had been taught an easy-to-follow algorithm that assists laypersons in performing CPR (check for consciousness, check for breathing, call for help, carry out chest compressions) [8]. Most participants adhered to this algorithm during the simulated scenario, regardless of whether the app was used or not. Check for consciousness was performed correctly by most; check for airway obstructions and assessment of normal breathing presented a bigger challenge. Even participants who received detailed instruction by the app had difficulties with this task. Video instructions were shown to be more helpful than audio-guidance for implementing proper airway management (head tilt/chin lift manoeuvre and check for breathing) [33]. The audio-visual guidance provided by the app used in our study may have lacked the necessary clarity to significantly improve the participants’ performance in this task.

High-quality chest compression is essential for surviving a cardiac arrest [34]. A rate of 100–120 compressions per minute was shown to be the optimal frequency [35, 36]. When instructed by the app (mandatory group), participants significantly more often performed chest compressions within the recommended pace. This might be accountable to the metronome-function of the app, as has also been suggested by Paal et al. [37].

High-quality CPR requires a chest compression depth of 5–6 cm [36]. In our study, participants in the mandatory group achieved the recommended compression depth significantly more often. This advantage of the app seems to be independent of how the instructions are presented, since studies that used audio-guidance as well as studies that used video-guidance have reported an improvement in compression depth [32, 33].

A total release of compression is also necessary [31]. We found no significant difference between the study groups regarding the release of chest compression, probably due to the fact that the app does not address this task. Also, no significant differences were found regarding the hand position on the chest.

The correct body position and arm posture (kneeling; both arms stretched) greatly facilitates performing adequate chest compressions. Participants with app-guidance all had a correct body position; correctness of arm posture was similar in all groups. Overall, the quality of body and arm positioning was high - probably because of the hands-on CPR-training six weeks before. Since psychomotor skills are best taught in direct training with manikins [33], smartphone-guidance should only be considered an additional tool to traditional hands-on CPR training.

Considering that resuscitation in a cardiac arrest has to be initiated as soon as possible [7, 31, 38, 39], it is of paramount importance that using a smartphone app does not lead to substantial time delay. We found that participants who were instructed to use the app took longer time to start the check for breathing, to call for help and to perform the first chest compression. This association has also been reported by other authors [25, 32, 37]. In studies comparing the mode of presentation, app-/or video-demonstrated instructions have led to a shorter delay than audio-only instructions (as in telephone-CPR) [40, 41]. Yet, out of all apps providing real-time guidance, only few are user friendly and in concordance with current resuscitation guidelines [30, 42]. Our findings underline that the usefulness of an app depends on its instructional quality, applicability and clarity. In our study, only half of the participants in the facultative group decided to use the app. While the mandatory group received an introduction to the app and practiced its operation during hands-on CPR-training, the facultative group only received a handout with details on how to download it. This difference in familiarization seems to be decisive for the actual use of the app. When access, instruction and encouragement are provided, an app is more likely to be used and to be perceived as helpful [43]. Incorporating the app into the syllabus of first aid courses might therefore increase its usage.

Medical laypersons without CPR-training tend to be overwhelmed and may prefer not to help rather than to do something wrong when witnessing an out-of-hospital cardiac arrest [44]. Smartphone guidance may prompt them to assess consciousness and breathing and helps bystanders with limited CPR knowledge remember all steps. In our study, the time delay caused by operating the app was approximately one minute. This is a relatively small loss of time compared to a situation, in which no bystander CPR is initiated, and the first chest compression is not performed until the ambulance crew arrives. In situations, in which the ambulance takes 10 min or longer to reach the emergency site, the initial time delay might be mitigated by a shorter overall hands-off time and an improved quality of bystander CPR.

### Limitations

This study is a simulation study with manikins, thus, generalisability to real-life scenarios is limited. Resuscitation was performed with one-person hands-only CPR, which might be different in real-life scenarios. This study was done with pupils. Because of their young age, transferability to other bystanders may be reduced. Participants in the mandatory group were slightly older than in the other groups, which could lead to a greater body strength and deeper chest compressions [45]. However, height and weight differences in pupils aged between 14 and 15 years old are not as pronounced as in younger years [45].

School pupils were chosen as study participants, because they have comparable general knowledge, comparable knowledge regarding medical emergencies and resuscitation and comparable physical fitness. Additionally, school kids use internet and apps as their main source of information and first choice in cases of questions [46, 47], hence are bound to be more likely to use a smartphone app. This study population includes both persons interested and not interested in CPR; in contrast to studies, which recruited volunteers out of the general public [48]. People responding to a call for a study might have a higher motivation than the ones not responding, leading to a bias.

Allocation into the three groups was done on school level to reduce communication between different study groups. However, it bears the risk of bias by different educational strategies of different schools. We tried to diminish this bias by selecting the same school type and by providing every participant with a standardised CPR-training.

The timespan between training and examination was six weeks. Other results may have been observed after a different time interval or with laypersons without previous CPR-training. Participants in the mandatory group were trained on how to operate the app. Outside the study environment people often download apps without familiarizing with it [49]. Thus, they might not know how to operate the app in case of witnessing cardiac arrest, which could lead to longer time delays before starting CPR.

During the simulated scenario, chest compressions were stopped after two minutes to allow for standardisation, similar to other studies [25, 50]. In real emergencies, bystander CPR is often required for a longer time-interval, probably pronouncing or mitigating the differences in quality of chest compressions.

### Future outlook

The advantages of app-guidance on the quality of bystander CPR have to be confirmed in real-life cardiac arrests before a final recommendation can be made. The future goal is to analyse the impact on return of spontaneous circulation and quality of life.

## Conclusions

While some aspects of high-quality CPR were improved when the smartphone app was used (correct chest compression rate and depth), other aspects remained a challenge to medical laypersons (assessment of airway obstruction and normal breathing). Operating the app caused a delayed start of chest compressions and resulted in a longer total hands-off time. Provided that the app gives easy-to-implement, guideline-compliant instructions and that the user is familiar with its operation, we recommend smartphone-guidance as an additional tool to hands-on CPR-training to increase the prevalence and quality of bystander-initiated CPR.

## Supplementary information

**Additional file 1.**

**Additional file 2.**

## Data Availability

The datasets generated and analysed during the current study are available from the corresponding author on reasonable request.

## Abbreviation

App: Smartphone application
CPR: Cardiopulmonary resuscitation
