# Supplementary material for: Smartphone apps to support laypersons in bystander CPR are of ambivalent benefit: a controlled trial using medical simulation
Source: Scand J Trauma Resusc Emerg Med. 2021 Jun 3;29:76. doi: 10.1186/s13049-021-00893-3 (PMC8173850; doi:10.1186/s13049-021-00893-3)
Supplement: Supplementary file 1 — Additional file 1. [file 13049_2021_893_MOESM1_ESM.pdf]

Score chart	number:

Check:

Assessment of consciousness:

- ☐ Not checked
- ☐ Spoke to or touched person
- ☐ Spoke to and touched person

Assessment of airway obstruction:

- ☐ Not checked
- ☐ Checked, but not correctly
- ☐ Correctly checked

Assessment of breathing

- ☐ Not checked
- ☐ 1 out of look, listen, feel
- ☐ 2 out of look, listen, feel
- ☐ 3 out of look, listen, feel

Call:

- ☐ No call for help
- ☐ Called for help
- ☐ Called 112 or let someone else call 112
- ☐ Called 112 or let someone else call 112 and called for help

Chest compression:

Body position:

- ☐ Not correct
- ☐ Correct

Arms hold straight?

- ☐ Not correct
- ☐ Correct

Time measurement:

	Time
Start of check of breathing	
End of check of breathing	
Call for help	
First chest compression	

Participants of facultative and mandatory group:

App used?

- ☐ No
- ☐ Yes

App worked without problems?

- ☐ No
- ☐ Yes

If the participant didn´t do chest compressions:

What´s the participant doing?
