# Supplementary material for: Smartphone apps to support laypersons in bystander CPR are of ambivalent benefit: a controlled trial using medical simulation
Source: Scand J Trauma Resusc Emerg Med. 2021 Jun 3;29:76. doi: 10.1186/s13049-021-00893-3 (PMC8173850; doi:10.1186/s13049-021-00893-3)
Supplement: Supplementary file 2 — Additional file 2. [file 13049_2021_893_MOESM2_ESM.docx]

E-Table 1: Measured time intervals during simulation

|  | **Control group (%)** | **Facultative group (%)** | **Mandatory group (%)** |
| --- | --- | --- | --- |
| **Time until check for breathing (sec.)** |  |  |  |
| mean (standard deviation) | 12.2 (±8.2) | 19.7 (±14.1) | 23.1 (±9.4) |
| median (lower; upper quartile) | 10 (7.5; 13) | 15 (10; 26) | 17,5 (12; 23.5) |
| **Time to check breathing (sec.)** |  |  |  |
| mean (standard deviation) | 6.6 (±3.7) | 8.5 (±7.2) | 7.9 (±4.3) |
| median (lower; upper quartile) | 6 (4; 9) | 6 (4; 9) | 7 (5; 10) |
| **Time until call for help (sec.)** |  |  |  |
| mean (standard deviation) | 19.5 (±14.8) | 44.6 (±36.2) | 35.2 (±13.7) |
| median (lower; upper quartile) | 17 (12; 22) | 30 (24.3; 57.8) | 32 (26; 42) |
| **Time until first compression (sec.)** |  |  |  |
| mean (standard deviation) | 29.2 (±12) | 51.1 (±29.5) | 68.1 (±19.4) |
| median (lower; upper quartile) | 28 (20; 36) | 42 (28.8; 65.8) | 66.5 (54; 77.3) |
| **Hands-off time during compression (sec.)** |  |  |  |
| mean (standard deviation) | 1.5 (±5.5) | 11.0 (±19.3) | 0.5 (±1.6) |
| median (lower; upper quartile) | 0 (0; 0) | 0 (0; 14) | 0 (0; 0) |
| **Total hands-off time (sec.)** |  |  |  |
| mean (standard deviation) | 31.2 (±13.2) | 61.1 (±33.9) | 67.8 (±19.1) |
| median (lower; upper quartile) | 29 (21; 36.8) | 53 (34.5; 80.5) | 65,5 (55; 77) |
